# Is China Moving toward Healthy Aging? A Tracking Study Based on 5 Phases of CLHLS Data

**DOI:** 10.3390/ijerph17124343

**Published:** 2020-06-17

**Authors:** Yinan Yang, Yingying Meng

**Affiliations:** 1 School of Public Administration, South China University of Technology, Guangzhou 510641, China; 2 Centre for Social Security Studies, Wuhan University, Wuhan 430072, China

**Keywords:** health, comprehensive assessment, latent variable, second-order factor model, structural equation modeling

## Abstract

Health is the key to the aging problem, and “healthy aging” depicts the overall changing trends in the health of all elderly individuals in a society. Based on the Chinese Longitudinal Healthy Longevity Survey (CLHLS) data from the years 2002, 2005, 2008, 2011 and 2014, this article investigates whether there is a trend of “healthy aging” in China. A second-order factor model including four dimensions of physical health, functional status, mental health and social health was constructed to measure a latent variable, “Health_elders”. The further multigroup comparison results by structural equation modeling showed that, with the exception of 2008, the Health_elders in 2002, 2005, 2011 and 2014 displayed an upward trend, and the mean differences in Health_elders across five periods were significant. These findings indicate that on the whole, compared with older people in the past, older people in more recent periods are healthier, which supports the trend of “healthy aging” in China. In terms of cohorts, the average health levels of male, town-residing elderly populations are higher, while the healthy aging trends among female, rural and urban elderly populations are stronger. Moreover, the physical health levels of the 60–74 years-old cohort are decreasing, and the participation of elderly individuals in social activities is low, which are the weaknesses in the healthy aging process in China.

## 1. Introduction

Health is the key to the aging problem, and solving health problems can essentially resolve the negative impact of aging [1,2]. Havighurst first proposed the concept of “healthy aging” [3]. In 1990, the first World Assembly on Aging officially introduced the strategy of “healthy aging” to its member nations. The strategy called for enhancing or maintaining the internal abilities of elderly individuals and improving supportive environments to realize the functions required for the healthy life of elderly people [4]. China has the largest number of elderly people in the world, and some scholars have pointed out that healthy aging is the key way for China to cope with the challenges of population aging [5,6,7].

“Healthy aging” highlights the overall changing trends in the health of all elderly individuals in a society [1,8]. China was recognized as an aging society in 1999, and by 2019, elderly individuals over 60 years old accounted for 18.1% of the total population and those over 65 years old accounted for 12.6%. In the past 20 years, has China moved towards healthy aging, sub-healthy aging or even diseased aging? According to the Healthy China Action Promotion Committee, in 2018, China’s average life expectancy was 77 years, of which the healthy life expectancy was 68.7 years; that is, elderly individuals lived with diseases for approximately 8.3 years, including 40 million half-disabled elderly individuals and 20 million completely disabled elderly individuals, and 180 million elderly people with one or more chronic diseases, accounting for 75% of this population. In addition to the above officially disclosed data, researchers have also carried out a rich exploration and assessed the changing trends in the health status of the elderly population in China. Some of them have found that the elderly population is getting healthier [9,10,11], while some have reached opposite conclusions [12,13,14]. In addition, a study obtained positive results in a few indicators and negative in other indicators, without consistent results [15].

The annual statistical data and research studies are primarily based on cross-sectional data in a certain year. These measurement results can only reflect the static level of elderly health at that time point and cannot describe the long-term changes. Individual longitudinal research studies based on multistage data often compare only the measurement results in each stage but do not test the significance of the differences between measurement values across the periods. Therefore, whether the changes across time periods show statistical significance has not been tested. Most of the measurement tools use one or several indicators, which are often measured separately. A single or a few indicators are not enough to reflect the whole picture of the health of the elderly population, leading to one-sidedness and uncertainty [16]. However, due to different research methods and variables used in the construction of indicator systems, most of the results lack comparability [17,18]. These shortcomings obviously affect the accurate understanding of the overall characteristics and trends in aging health in China and may be the main cause of the inconsistencies in the existing empirical results.

In this paper, improvements over previous studies is made. Using five phases of longitudinal data, the “overall health” of elderly individuals is measured with four dimensions of physical health, functional status, mental health and social health. Thereafter, statistical methods are performed to test the significance of difference across the five-phase measurement results of average health in the elderly population. Finally, the trends in healthy aging in China are judged based on the results of these dynamic measurements and tests.

## 2. Literature Review

In response to the challenges of population aging, Havighurst first proposed the concept of “healthy aging” [3], which was defined as prolonging life span and increasing life satisfaction. However, longevity reflects only the quantity of life, not the quality of life. In 1987, the World Health Assembly extended the definition of “healthy aging” as postponing biological aging and social aging through a series of positive measures whilst unstoppable calendar aging continues. Rowe and Kahn defined healthy aging as an “active disease-free state”. These definitions ignore the inevitable aging process in the process of life, and it is not realistic to expect elderly individuals to have no disease [19]. In view of this, in 2016, the World Health Organization no longer emphasized the lack of “disease” in its Global Report on Aging and Health but defined “healthy aging” as “the process of developing and maintaining the functions required for healthy life of the elderly” based on the perspective of “function” [4]. This definition mainly depends on older people’s internal abilities, supportive environment and their interaction.

The concept of “healthy aging” based on functional performance is widely accepted, but its measurement indicators, statistical analysis methods and models are still very limited [20,21,22]. This is because measuring healthy aging is not simply equivalent to measuring the health of elderly individuals. There are two large differences in measuring healthy aging: first, the health of elderly individuals is the result of the gradual development of people across the whole life process [23]. Individuals’ choices at different time points and environmental interventions during the life process will change their internal abilities and function and ultimately affect the changing trajectory of healthy aging [24]. Therefore, we should dynamically investigate or intervene in healthy aging based on the whole life course [4]; second, although healthy aging should be based on individual’s health and longevity, it focuses on the overall situation or average level in the elderly population [25]. The healthy life span of a few people has little effect on improving the average healthy life span of the group [26]. Xiong and Dong [27], Wu and Jiang [1] and Zhong and Chen [28] pointed out that “healthy aging” can be considered from two aspects: first, healthy life expectancy is increasing, the corresponding period of disease is shortening, and the best goal is to “end without disease”; second, the proportion of healthy and long-lived elderly people accounts for the majority and is increasing. It can be seen that healthy aging is based on the measurement of the overall health level of the elderly population and then the trends in the dynamic changes.

The most commonly used indicators of elderly health include life expectancy, health expectancy, quality-adjusted life expectancy (QALE), disability-adjusted life expectancy (DALE, which was renamed HALE in 2001), years of life lost (YYL), incidence rate, disability rate and other indicators [29]. In research studies, some researchers will use a single indicator, while others will use multiple indicators. It is difficult to reflect the overall health status of elderly individuals with only a single indicator or a single dimension. Measurement errors often lead to the illusion that elderly individuals suffer from group diseases or functional problems [18,30]. Therefore, to date, researchers have measured the health of elderly individuals from three dimensions of physical health, mental health and social health according to the definition of health from the World Health Organization [29]. When elderly individuals are asked about themselves, they think that healthy aging should include four aspects: physical, mental, psychological and social [31]. These dimensions are usually measured using the activities of daily life (ADL) scale, instrumental ADL (IADL) scales loneliness scales, the Mini-Mental State Examination (MMSE), and social relationship scale (SRS), etc. [32].

In recent years, more emphasis has been placed on the comprehensive evaluation of the health of elderly individuals based on the concept of overall health including physiological health, physical function, mental health, role function, self-assessment health and other dimensions, as well as health-related quality of life assessment (HRQOL), comprehensive geriatric assessment (CGA), etc. [33,34]. A developed comprehensive assessment scale includes the Duke University Older Americans Resources and Services (OARS) assessment scale, the Comprehensive Assessment and Referral Evaluation (CARE), the Frailty Index (FI), and the Grade Membership of Health Status [29]. Compared with traditional measurement methods, the comprehensive assessment of elderly health as a predictor of elderly health is much better, which can provide a more appropriate entry point for public health policy [4,18].

It can be seen that the measurement of elderly health has experienced a development process from a single indicator to multiple indicators and from a single dimension to multidimensional to a comprehensive evaluation. The same process also stands for the research on the measurement of elderly health in China. Zhong and Chen [28] and Qiang and Zhe [35] used the Sullivan method to measure the healthy life expectancy of Chinese elderly individuals. Wu et al. [36] proposed measuring the health of Chinese elderly individuals with three indicators: life span, self-care ability evaluations and self-assessed health. Wu. Y and Dang [37] showed that about 50% of older people in China experienced noncommunicable diseases; and more than 37 million had significant reductions in physical function. Rao [5] selected four indicators to measure the social effect of healthy aging, including the Chinese elderly individuals’ life independence, spiritual pleasure, social interaction and social contribution. In fact, these studies all use single indicator for the measurement. Jiao [18] made a contribution in measuring the overall health of elderly individuals by using the ADL, IADL, MMSE and self-reported measures of chronic diseases and used a latent class model to classify the Chinese elderly participants into four categories: healthy, mild disability, cognitive impairment and mostly unhealthy. Based on the four dimensions of life independence, spiritual pleasure, social interaction and participation, and social contribution, Qian [38] found that China’s healthy aging has achieved some results, but there is still much room for improvement, especially in the health status of the elderly population in rural areas. Smith et al. [39] used a cross-sectional data of China Health and Retirement Longitudinal Study 2011 baseline sample, and found that both cognitive and physical health exhibit strong negative age patterns. These researchers have tended to understand healthy aging as a measurement of aging health and attempt to reflect trends in healthy aging from the results of some cross-sectional data. Clearly, the static measurement results have difficulty reflecting long-term trends in healthy aging in China.

Some researchers have realized that the trends in healthy aging in China should be judged from the perspective of dynamic change. Mu [40] proposed a set of eight indicators including the change in proportion of healthy elderly in the total population and the change in proportion of healthy elderly in the total elderly population to reflect health changes in the elderly population, but the data were not used to conduct specific measurement. Zhe, Xiang and Fang [12] used ADL scores to track 12-year survey data from Beijing elderly individuals, which showed that the ratio of healthy life expectancy (ALE/LE) decreased in recent years. A study from the World Health Organization found that the gap between the average life expectancy and the healthy life expectancy of elderly individuals in China increased with increasing age in the period from 2000 to 2012 [41]. Zeng and Feng [14] showed that cognitive function and physical function of Chinese elderly individuals significantly decreased from 1998 to 2008 compared with 10 years ago. The increase in life expectancy per capita was accompanied by a decline in the health level of elderly individual. In contrast to the conclusions of these studies, other studies found that Chinese elderly individuals are becoming healthier [9,10,11]. Yu and Feng [15], based on the data of the China Nutrition and Health Survey (CHNS) in the period 1991–2009, using a random effect model, found that indicators of daily behavioral abilities gradually improved between generations, but the indicators of chronic disease and health risk gradually deteriorated and was more severe in rural areas. Zhang and Wang [42] employed data from the China’s population census in 2010 and the 1% population sample survey in 2015, the results show that the health status of the urban elderly remained stable, but that of the rural elderly improved between the years 2010 and 2015. The prevalence of disability of the elderly declined between 1994 and 2015. These studies have focused on dynamic measurements of healthy aging, but their shortcomings are that they also examined a single health indicator, or a few indicators, and the empirical results were not conclusive.

From the perspective of measurement research on healthy aging in China, the existing problems are that the measurement indicators are too few, the dimensions are single, and the results are not consistent. In addition, most of these studies were aimed at static measurements of cross-sectional data at a particular period of time or lack statistical tests assessing the significance of difference between measures across multiple periods of data. These shortcomings have limited judgments regarding trends in healthy aging in China.

## 3. Study Design

### 3.1. Data

The data used in this paper are from the Chinese Longitudinal Healthy Longevity Survey (CLHLS). The project covers 23 provinces and is the largest set of survey data of the elderly population in China. This paper selects five longitudinal datasets from 2002, 2005, 2008, 2011 and 2014. Among them, 16,064 observations were made in 2002, with an average age of 86, 15,638 in 2005 in which 8175 observations were interviewed in the former waves and 3898 were newly added, with an average age of 86, 16,445 in 2008 in which 7475 observations were interviewed in the former waves and 6038 were newly added, with an average age of 87, 9749 in 2011 in which 8423 were interviewed in the former waves and 647 were newly added, with an average age of 86, and 7168 in 2014 in which 6067 were interviewed in the former waves and 1125 were newly added, with an average age of 85. We removed 135 individuals who were under 60 years old from the sample and finally obtained 65,064 observations, with the minimum of 60 years old and the maximum of 120 years old.

### 3.2. Health Dimensions and Measurement Indicators

The CLHLS data investigated the health status of elderly individuals from multiple dimensions. The measurements are included the following Table 1:

To reflect the overall health of the elderly population and reduce the measurement error, this paper selected all four dimensions and nine items in Table 1. Among them, functional status, mental health and social health were all positive measures of the health of elderly individuals, while physical health were reverse measures of the health of elderly individuals.

### 3.3. Measurement Model and Method

#### 3.3.1. Second-Order Factor Model

According to the definition of health from the World Health Organization, it is composed of four dimensions: functional status physical health, mental health and social health. Therefore, a second-order factor model was appropriate to measure the health of the elderly population. The second-order factor model believes that there is a common second-order factor that explains the correlations between the first-order factors. That second-order common factor is Health_elders which was measured by the four first-order factors. The matrix equation of the second-order factor model is as follows:

(1) η=Γξ+ζ

(2) $$X=\Lambda_{x}\xi +\varepsilon$$

**Figure 1:**
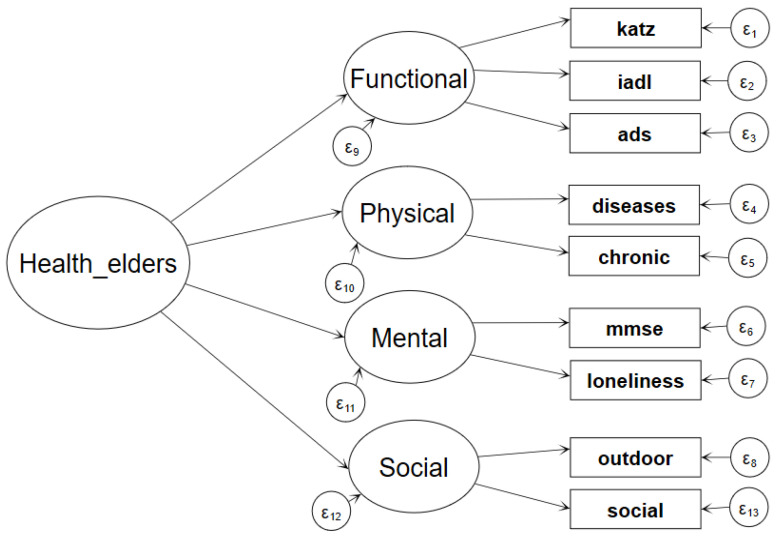
Theoretical Two-Order Factor Model.

Equation (1) is the second-order factor measurement model, *η* is the second-order factor vector, Health_elders, which is a continuous latent variable obeying a normal distribution. *ξ* is the first-order factor for Health_elders (functional status, physical health, mental health, social health), γ is the load coefficient of four first-order factors in the second-order factor, and *ζ* is the second-order factor residual. Equation (2) is the measurement model of the four first-order factors *ξ*, *X* is the vector of the nine indicator variables. Λ*_x_* is the load coefficient of the nine indicators in the four first-order factors, and ε is the measurement error of indicator variables. The specific model is shown in Figure 1.

#### 3.3.2. Significance Test of the Latent Variable’s Mean Difference Using a Multiple-Group Comparison Sturctural Equaiton Modeling

After the second-order factor, Health_elders, is properly measured and the model was estimated and accepted, we continue to examine the health aging trend in the five periods. The intercept of the second-order factor model in Equation (1) is fixed to be 0 by default. However, it will be set as free parameter to be estimated by fixing the variance of the latent variable Health_elders to 1 in the structural equation model. We then utilize the structural equation model’s function of multiple-group comparison to test the differences significance of the Health_elder’s mean across five stages. The matrix equation is shown in Equation (3):

(3) η=μ+Γξ+ζ

The structural equation model in Formula (3) can estimate the new parameter, namely, the latent variable mean *μ*. When comparing multiple groups, it will set the latent variable mean value of the reference group to 0 and then test whether the difference between the mean value of the other groups and the reference group was significant [43]. We conducted four tests for 2002–2005–2008–2011–2014 (base group 2002), 2005–2008–2011–2014 (base group 2005), 2008–2011–2014 (base group 2008) and 2011–2014 (base group 2011) to observe whether the changes in latent variable mean values in each group were significant and to study and judge the trends in the changes regarding healthy aging.

## 4. Empirical Results

### 4.1. Descriptive Results

The descriptive statistical results of the nine indicators are shown in Table 2.

Table 2 uses the summary data from the five phases. We also calculated the average trend in the nine health indicators over the five periods of data. In general, the five health indicators of ADL, IADL, ADS, MMSE and loneliness had high five-stage total mean values. The average values across the five periods rose, which showed that the functional status and mental health of elderly individuals were improving.

Regarding the physical health indicators, the total mean scores across the five stages for chronic diseases was 1.12, and the total mean scores across the five stages for serious diseases over the previous two years was 0.28. Among them, the number of chronic diseases was 1.15 in 2002, 1.21 in 2005, 1.0 in 2008, 1.15 in 2011 and 1.13 in 2014. In the previous two years, the number of serious diseases was 0.24, 0.28, 0.24, 0.35 and 0.38 in these five periods, respectively, showing a slight upward trend in general. In terms of the ratios, the ratio of patients with more than one chronic disease was 61.66% in 2002, 61.65% in 2005, 56.63% in 2008, 58.86% in 2011 and 60% in 2014 (the average of the five periods was 60%). The incidence of more than one serious disease was 12.45% in 2002, 14.35% in 2005, 11.76% in 2008, 14.37% in 2011 and 14.5% in 2014 (the average of the five periods was 13.23%). This is close to the results of Guo et al. (2019) using the fifth national health service survey data in 2013 [44]. They found that the prevalence of chronic diseases in elderly individuals was 56.4%, and the annual hospitalization rate was 16.8%. According to the data released by the Health China Action Promotion Committee, 75% of elderly individuals suffered from one or more chronic diseases in 2018.

The total average value for the five periods regarding social activities was 1.28, which corresponded to “occasionally participate”, and this value was low. However, the average value across each period slightly increased, indicating that the participation of elderly individuals in social activities has been improving. The number of outdoor activities for elderly individuals slightly decreased, but the total average value for the five periods was still 3.14, corresponding to “at least once a month”, which was generally optimistic. Although regular moderate physical activity can delay the decline in physical function, a high proportion of the elderly live an inactive life in most countries [45]. Li and Gao [46] found that the self-organization of social participation of elderly individuals in Chinese cities was not high, and the passive tendencies in the willingness and ways to participate were very obvious. The fifth survey on the living conditions of elderly individuals in urban and rural areas in 2015 showed that 49.4% of elderly individuals never exercise [47].

In general, six out of the nine indicators showed a dynamic upward trend from 2002 to 2014. However, the indicator of serious diseases showed a trend towards deterioration, while the number of chronic diseases has remained stable. The indicator of outdoor activities also slightly decreased.

### 4.2. Second-Order Factor Model Estimation Results

We used the CLHLS 2002, 2005, 2008, 2011 and 2015 data to estimate the second-order factor model in Equation (1). In particular, we used the structural equation model (SEM) in Stata to estimate the target factor model. The use of SEM has more advantages, such as isolating measurement errors to obtain better estimation coefficients and predicted values, reporting model fitting indicators, and conducting more follow-up tests [48]. The parameters were estimated by the maximum likelihood method and are reported in Table 3.

According to the fitting indices in the five periods data shown in Table 3, RMSEA (root-mean-square error of approximation), CFI (Comparative Fit Index), TLI (Tucker-Lewis Index) and SRMR (Standardized Root Mean Square Residual) were all above the acceptable critical values, and R^2^ exceeded 0.9, indicating that the second-order factor model could be statistically accepted. From the results of the parameter estimation, the load coefficients of the second-order factor to the first-order factors were all significant at the 1% level (the load coefficients of Functional Status were automatically set to 1 by the program), the load coefficients of the first-order factor to the index variables were also significant at the 1% level (the load coefficients of the first index were automatically set to 1 by the program), and the parameter estimators were relatively close, which showed that the measurement results were good. This showed that the measurement coefficients of the two-order factor model in the five phases were robust and met the basic requirements of measurement invariance [48]. The model can be used to measure the latent variable Health_elders in the five periods.

**Figure 2:**
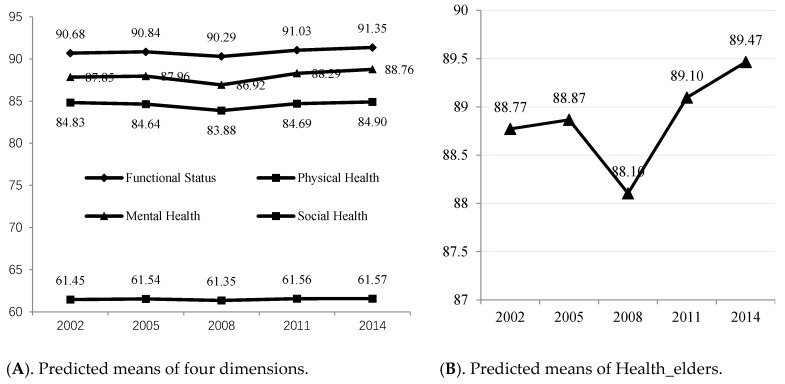
Predicted mean values of Health_elders and the four dimensions in five periods.

After the model fitting, we used the Stata’s predict command to estimate the prediction value of the second-order factor, Health_elders, and the four first-order factors. As these predicted values are standardized values, for ease of comprehension, we used the efficacy coefficient method to convert the predicted values to a 100-point scale. Figure 2 reports the predicted mean of the five latent variables.

According to Figure 2A, the predicted mean values for Functional Status, Mental Health and Social Health among the first-order factors continued to rise in the other four periods, with the exception of a decrease in 2008. The predicted mean value for physical health rose slightly (0.07 in value), but because physical health was measured in the reverse direction, indicating that elderly physical health was potentially in the risk of declining.

Figure 2B shows the predicted mean value of the second-order factor, Health_elders. With the exception of decrease in 2008, the overall trend towards increases across 2002, 2005, 2011 and 2014 showed an increase from 88.77 in 2002 to 89.47 in 2014. Compared with other years, the disasters in 2008 were more serious in China. At the beginning of 2008, there was a snow and rain disaster in the south, another Wenchuan earthquake with magnitude 8 in May, and a global subprime financial crisis in September. The GDP growth rate in that year dropped from 14.23% in 2007 to 9.65% in China. Zhu and Huang [49] used the CLHLS data to measure active aging, they also found that active aging in 2008 was at a low point. Using the “consistent index” compiled by the China Economic Climate Monitoring Center, they found that the trend in aging attitude was consistent with the macroeconomic environment. Elderly individuals are more dependent on the environment, but in the case of natural disasters, technological disasters and man-made conflicts, the elderly population is most likely to be ignored [4]. After 2008, elderly health in 2011 and 2014 proceeded to improve.

### 4.3. SEM’s Multiple-Period Comparison Test Results

Although the five-stage measurement results of latent variable Health_elders in Figure 2 demonstrate an overall upward trend, are the increases significant? If the elderly health value in each period increased, but the change was small but not significant, it would not be enough to support the conclusion that the elderly population in China is generally healthier. Therefore, we continued to use the structural equation modeling’s multiple-group comparison to test the mean differences in the five periods. When multiple groups were compared, the latent variable mean of the reference group was set to 0, and then the difference between the mean of other groups and the reference group were tested. We carried out four tests in total. The results are shown in Table 4.

In column (1) of Table 4, the average Health_elders in 2002 was taken as the base group (set as 0). The results showed that the mean value for Health_elders in 2005 was significantly higher (3.3%) than that in 2002. The mean difference in 2008 was negative but not significant. In 2011, it was significantly higher than 6.5% at the 1% level in 2002, while in 2014, it was significantly higher than 11.9% at the 1% level in 2002. Column (2) was based on the average Health_elders in 2005. The test results showed that in 2008, the level of 1% was significantly lower than that in 2005 by 4.9%. In 2011, it was 2.9% higher at 10% than in 2005, while in 2014, it was 8.2% higher at 1%. Column (3) was based on the average Health_elders in 2008. The test results showed that the level of 1% in 2011 was 7.8% higher than that in 2008, while the level of 1% in 2014 was 13.1% higher than that in 2011. Column (4) takes the average Health_elders in 2011 as the reference frame. The test results showed that in 2014, it was 5.5% higher than that in 2011. In Table 4, only 2008 was lower than 2002 (not significant), and 2008 was significantly lower than 2005.

In general, with the exception of 2008, the results from the other four periods showed that the average Health_elders in China is rising, and the rising change was statistically significant. Combined with the predicted trend in Figure 2B, it can be seen that compared with older people in the past, older people in more recent periods have better health levels. This strongly supports the notion that the trajectory of China’s aging is towards healthy aging. According to the fourth national survey on the living conditions of elderly populations in urban and rural areas, the proportion of self-rated elderly health in 2015 increased by 5.5% compared with that in 2000, including 1.4% in rural areas and 7.0% in urban areas [47]. Evidence from Fogel’s study of the United States also showed that older people do have better health than their grandparents and great-grandparents [50]. A pooled analysis by World Health Organization in 2014 of large longitudinal studies conducted in high-income countries suggested that the prevalence of severe disability was declining slightly, although no significant change in less severe disability has been observed during the past 30 year [51]. Oshio and Shimizutani [52] observes that the Japanese elderly’s additional work capacity has increased between 1986 and 2016 along with the improvement of health status.

### 4.4. Further Subgroup Results

**Figure 3:**
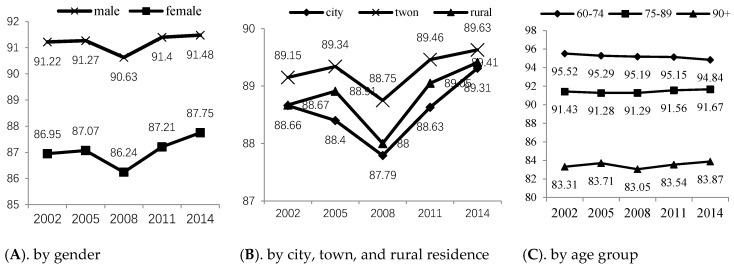
The predicted mean values of Health_elder in five periods.

We further divided the population by gender, urban, town and rural residential areas, and age groups to report the group differences underlying the overall trends. The results are shown in Figure 3:

Figure 3 shows that the average health predictions for the male and female elderly groups showed an overall upward trend, and the average health prediction for the male elderly group was higher than that for the female elderly group. However, from 2002 to 2014, the average health prediction for the female elderly had a larger increase, indicating that the healthy aging trend in the female elderly group was stronger. From the perspective of residential separation, the average health predictions for the elderly individuals in towns was higher; the second was the average health prediction for elderly individuals in rural areas, and the lowest was in the average health prediction for the elderly individuals in cities. Old people in rural villages had many problems, such as inconvenient access to medical treatment and medical and health resources. The elders in cities had to afford a high cost of living, bore greater psychological pressures, lived with fewer social interactions, and suffered great health loss from work. These reasons are also why an increasing number of old people are choosing to leave cities, especially large cities, to move to some characteristic counties and towns.

Regarding age cohorts, we divided elderly individuals by the ages suggested by the World Health Organization. The age groups were 60–74 years old (young elderly), 75–89 years old (middle-aged elderly), and over 90 years old (long-lived elderly). Among them, the health prediction values for the young elderly people was higher and that of the long-lived elderly people was the lowest. However, the health prediction values for the young elderly people decreased over the years. The young elderly people in 2014 were unhealthier than the young elderly people in 2002. This shows that with the faster pace of work and the greater pressures of life, the health loss in retired old people was greater than those of the same age in the past, resulting in the lower average value of group health. However, middle-aged and long-lived elderly people now have higher mean health predictions than those people of the same age in former periods. As previously demonstrated, old people in the past had better health endowments, coupled with better medical and health conditions and social security systems, the health status of the middle-aged and long-lived people is better than those in the past.

## 5. Discussion

This paper improves the existing measurement research on “healthy aging” from the perspective of overall health, with dynamic measurements over five periods of data testing for significant differences. The second-order factor model, which consists of nine indicators of functional status, physical health, mental health and social health, showed that, with the exception for lower levels in 2008, the predicted mean value of the other four phases of the latent variable Health_elders showed an upward trend and passed the significance test for the difference in the mean values of the latent variable. These results showed that the health status of the elderly population is generally on the rise, indicating that China’s aging is moving towards healthy aging. Compared with the older people in the past, older people are healthier now. This is due to obvious improvements in living conditions, such as better nutrition, medical and health services, and improvements in the health literacy of elderly individuals brought about by China’s economic and social development. In the past decade, China’s social security system also has been improved, including medical insurance, pension benefits, and pension service subsidies. In 2002, only 42.23 million old people were able to receive pensions, 24.74 million old people participated in medical insurance, and almost all the old farmers and residents had no pension or a very low-level pension. In 2014, 85.93 million retired employees were able to receive pensions, 143.13 million older people in urban and rural areas were able to receive pensions, 72.55 million retired employees participated in basic medical insurance, and 31.45 million urban residents were able to receive pension basic medical insurance (elderly individuals account for the majority of this population).

Zhe, Xiang and Fang [12], Zhang and Du [13], Zeng and Feng [14] and other researchers found that the elderly population in China becomes unhealthy is largely because they still regard disease-free as the judgment standard of health. The corresponding health indicators collected in their research were mostly limited to physiological health indicators such as disease, common disease, death, disability, and epidemiological methods [25]. However, health is multidimensional, and elderly health is more special and mainly includes noninfectious diseases such as chronic diseases, and many health problems that cannot be diagnosed as a certain disease, such as weakness, incontinence, falls, mental disorders, and syndromes, and often cannot be completely cured [4]. Even if these diseases exist, they cannot account for the actual impact on elderly individuals because these people can still maintain good function and enjoy a high level of health through drug inhibition, appliance assistance, environmental support, etc. [53]. For this reason, the latest definition of “healthy aging” from the World Health Organization no longer distinguishes healthy and unhealthy by the presence of disease but emphasizes the function exertion required in the healthy life of elderly individuals. It is believed that the effect of comprehensive evaluation results of function exertion in elderly individuals as the prediction index is much better [4]. This is also the reason why this paper chose a four-dimensional second-order factor model for comprehensive measurement.

The trend that the physical health dimension in elderly individuals tended to decrease deserves attention. China’s existing medical and health services are aimed at the treatment of acute infectious diseases and symptoms. The main characteristics of elderly patients are chronic diseases, comorbidities, physical deficiencies and so on. The course of disease is long, fluctuates with time, and is difficult to cure [4]. Although elderly individuals can also use the current medical and health system, it usually does not meet their needs well, and the effects are not good. However, the number of specialized medical and health institutions, rehabilitation hospitals and nursing homes for elderly individuals is limited and unevenly distributed. There is a serious lack of institutions such as dementia care and hospice care. The combination of medical and nursing services has just started, and the number of personnel engaged in elderly health services is also insufficient. If we continue to passively deal with these issues all the time, it will only increase the burden on the government and society and will fall into an increasingly passive situation [25]. As a matter of urgency, China should establish a relatively independent comprehensive elderly health service system including health care, prevention, treatment, rehabilitation, nursing care and hospice care as soon as possible, build a number of high-level specialized hospitals for elderly individuals, or take the second option and set up specialized geriatric departments in large-scale general hospitals, while providing professional teaching and training of gerontology and gerontology for health practitioner training and encouraging the medical and health system to adapt to the aging situation and requirements as soon as possible.

In addition, the study also found that the social participation of elderly individuals in China is low. Old age produces a change in a person’s social role, which inevitably leads to negative thoughts such as “useless”. Especially in rural areas, there are few social activities and few spiritual and cultural activities. China’s thirteenth five-year plan for the development of the national aging cause and the construction of an elderly care system proposes to “vigorously support the elderly to participate in social development”. The future policy should focus on developing voluntary services for elderly individuals, guiding the development of social organizations at the grassroots level, and actively promoting mutual support and pension models such as time banks, Internet plus pensions and smart pensions.

## 6. Conclusions

Health is the basic resource to ensure independence of and social participation among the elderly population. Healthy aging has changed people’s traditional view of aging, which can create rich new opportunities for elderly individuals, families and society [4]. In the future, the elderly population in China will exceed one third of the total population, account for more than a quarter of the whole life cycle, and account for more than 40% of the total medical expenses of the whole population [5]. This fact has determined a focus on the construction of “healthy China” is the health of the elderly population. Without “healthy aging”, it is impossible to achieve “healthy China”.

Is China’s aging healthy? This is a very important issue for system reform, optimization and policy implementation. In this paper, the estimation results showed that the health status of the elderly population is generally on the rise, indicating that China’s aging is moving towards healthy aging. Compared with the older people in the past, older people are healthier now. However, the measurement results from five stages of the CLHLS data showed that the proportion of chronic diseases in the elderly population in China has been as high as 60%, and physiological health indicators such as chronic and serious diseases have been slightly exacerbated. In addition, the study also found that the social participation of elderly individuals in China is low. More targeted policies are encouraged to help the Chinese elders to improved their physical health and social participation.

Finally, limited by the variables in CLHLS data, only nine indicators are used in the four dimensions measurement in this study. In particular, the physical health, mental health and social health can only be measured by two indicators. If more indicators can be obtained, the test results of China’s healthy aging trend will be more accurate.

## Figures and Tables

**Table 1:** Four Health Dimensions and Measurement Indicators.

Health Dimension	Indicator Variables
Functional status	ADL: six items, reflecting the abilities of self-care, including bathing, dressing, indoor activities, going to the toilet, eating, and controlling urination and defecation. Three points were given to those who did not need help, 2 points were given to those who needed help for one part, and 1 point was given to those who needed help for more than two parts. The scores of the 6 items of respondents were summed with a maximum of 18 points and a minimum of 6 points. IADL: Eight items, including whether the individual could visit their neighbor’s house, lift 5 kg of weight, wash clothes, cook, etc. Three points were given to those who could do it, 2 points were given to those who had certain difficulties, and 1 point was given to those who could not do it. The scores for the 8 items of the respondents were summed with a minimum of 8 points and a maximum of 24 points. ADS: six items measuring body function limitations: whether the hand could touch the back waist, whether the hand could touch the neck root, whether the individual could stand up from the chair, whether the individual could pick up a book from the ground, the steps required to rotate in situ, and whether the arm could be lifted. Completing the task with both hands received 3 points, one hand received 2 points, and neither hand received 1 point. After summing the scores, the maximum value was 18 points, and the minimum value was 6 points.
Physical health	Diseases: Number of serious diseases in the previous two years. Serious disease referred to the need for hospitalization or being bedridden at home. Chronic: Number of chronic diseases; each elderly person was required to report whether they had the listed chronic diseases.
Mental health	MMSE: Measured cognitive function in elderly individuals and included five aspects: orientation ability (general ability), response ability, attention and calculation abilities, recall ability, and language, understanding and self-coordination abilities, for a total of 24 questions. Seven points were given to the question, “How many food names can you say in one minute?” One point was given to the other 23 correctly answered questions, and 0 points were given to the incorrectly answered questions. The total score was 30. Loneliness: the elders were asked, “Do you often feel lonely?” Values of 1–5 represented always, often, sometimes, rarely and never.
Social health	Outdoor: Participation in outdoor activities, values 1–5 represent never, occasionally, at least once a month, at least once a week, and almost every day. Social: Participation in organized social activities, values 1–5 represent never, occasionally, at least once a month, at least once a week, and almost every day.

**Table 2:** Descriptive statistics of indicator variables (five periods pooled data).

Variables	Frequency	Mean	S.D.	Minimum	Maximum
ADL	64,317	16.89	2.490	6	18
IADL	64,770	18.05	6.100	8	24
ADS	64,025	15.76	2.880	6	18
MMSE	63,946	20.67	8.720	1	30
Loneliness	57,542	3.940	1.010	1	5
Outdoor	64,839	3.140	1.830	1	5
Social	64,812	1.280	0.820	1	5
Chronic	65,055	1.120	1.340	0	21
Diseases	63,465	0.280	0.780	0	30

**Table 3:** Second-order factor model estimation results.

Dimensions and Indicators		(1)	(2)	(3)	(4)	(5)
		2002	2005	2008	2011	2014
Elderly Health (second order)	Functional Status	1.000	1.000	1.000	1.000	1.000
	Physical Health	−0.078 ***	−0.089 ***	−0.063 ***	−0.044 ***	−0.068 ***
		(−13.28)	(−14.47)	(−10.23)	(−3.44)	(−4.44)
	Mental Health	0.195 ***	0.196 ***	0.191 ***	0.204 ***	0.170 ***
		(21.65)	(20.25)	(20.16)	(16.77)	(11.77)
	Social Health	0.192 ***	0.221 ***	0.180 ***	0.203 ***	0.195 ***
		(26.24)	(26.26)	(23.31)	(18.81)	(14.44)
Functional Status	ADL	1.000	1.000	1.000	1.000	1.000
	cons.	17.174 ***	17.293 ***	17.420 ***	17.232 ***	17.310 ***
		(1155.00)	(1131.16)	(1209.35)	(829.82)	(703.74)
	IADL	4.906 ***	4.664 ***	4.920 ***	4.518 ***	4.706 ***
		(107.83)	(98.21)	(97.07)	(72.01)	(61.23)
	cons.	18.726 ***	18.952 ***	18.970 ***	19.181 ***	19.404 ***
		(406.73)	(404.59)	(400.27)	(311.01)	(257.15)
	ADS	1.493 ***	1.506 ***	1.796 ***	1.366 ***	1.444 ***
		(83.74)	(85.77)	(89.81)	(63.22)	(54.26)
	cons.	16.197 ***	16.199 ***	16.074 ***	16.322 ***	16.323 ***
		(831.41)	(806.63)	(758.67)	(647.92)	(523.72)
Physical Health	Serious illness	1.000	1.000	1.000	1.000	1.000
	cons.	0.233 ***	0.269 ***	0.228 ***	0.351 ***	0.398 ***
		(41.07)	(47.71)	(40.02)	(30.16)	(27.96)
	Chronic disease	2.334 ***	2.084 ***	1.317 ***	1.542 ***	0.823 ***
		(11.90)	(12.24)	(7.17)	(3.00)	(2.77)
	cons.	1.126 ***	1.177 ***	0.982 ***	1.176 ***	1.185 ***
		(101.44)	(99.68)	(96.43)	(74.41)	(62.64)
Mental Health	Lonely feeling	1.000	1.000	1.000	1.000	1.000
	cons.	3.933 ***	3.959 ***	3.939 ***	3.985 ***	3.945 ***
		(459.47)	(458.21)	(453.11)	(368.46)	(302.53)
	MMSE	20.444 ***	22.208 ***	21.856 ***	21.899 ***	24.200 ***
		(21.96)	(20.57)	(20.20)	(16.94)	(11.91)
	cons.	22.752 ***	22.956 ***	22.341 ***	23.046 ***	23.523 ***
		(439.16)	(412.91)	(428.45)	(316.10)	(278.18)
Social Health	Social activities	1.000	1.000	1.000	1.000	1.000
	cons.	1.299 ***	1.333 ***	1.278 ***	1.310 ***	1.330 ***
		(185.78)	(174.80)	(181.33)	(132.93)	(106.89)
	Outdoor activities	5.155 ***	4.290 ***	4.780 ***	4.399 ***	4.538 ***
		(25.96)	(26.29)	(23.30)	(18.45)	(14.41)
	cons.	3.441 ***	3.304 ***	3.224 ***	3.256 ***	3.243 ***
		(233.20)	(221.89)	(212.26)	(159.01)	(128.81)
Goodness of fit indices	N	14149	13855	13961	7860	5216
	RMSEA	0.056	0.058	0.047	0.049	0.044
	CFI	0.968	0.965	0.975	0.974	0.980
	TLI	0.951	0.945	0.960	0.959	0.968
	SRMR	0.055	0.044	0.031	0.047	0.039
	R^2^	0.943	0.907	0.966	0.909	0.916

Note: The z value is reported in parentheses, *** represents a significance level of 0.1%.

**Table 4:** Significance test of the latent variable’s mean differences using a multiple-group comparison structural equation model.

Results	Period	Five Periods	Four Periods	Three Periods	Two Periods
Mean differences test of Health_elders(latent variable)	2002	0.000			
	2005	0.033 **	0.000		
		(2.41)	(.)		
	2008	−0.011	−0.049 ***	0.000	
		(−0.80)	(−3.45)	(.)	
	2011	0.065 ***	0.029 *	0.078 ***	0.000
		(4.03)	(1.80)	(4.89)	(.)
	2014	0.119 ***	0.082 ***	0.131 ***	0.055 ***
		(6.41)	(4.38)	(7.04)	(2.65)
Measurement model estimation results	second order	omitted	omitted	omitted	omitted
	first order	omitted	omitted	omitted	omitted
Goodness of fit indices	N	55,041	40,892	27,037	13,076
	RMSEA	0.064	0.063	0.062	0.057
	CFI	0.934	0.937	0.939	0.953
	TLI	0.931	0.933	0.933	0.945
	SRMR	0.055	0.051	0.048	0.055
	R^2^ (CD)	0.920	0.917	0.922	0.931

Note: The z value is reported in parentheses; ***,**,* represents a significance level of 1%, 5% and 10%.
